# Extracorporeal Carbon Dioxide Removal (ECCO₂R) as a Bridge to Extracorporeal Membrane Oxygenation (ECMO) in Severe H1N1-Associated Acute Respiratory Distress Syndrome (ARDS) in Morbid Obesity: A Case From a Resource-Limited Hospital

**DOI:** 10.7759/cureus.103955

**Published:** 2026-02-20

**Authors:** Julio A Velandia-Escobar, Claudia H Mojica-Castañeda, Juan D Bustos-Acosta, Yardany R Méndez-Fandiño, Jonathan Alexander Guezguan-Perez, Liliana P Acero-Galvis

**Affiliations:** 1 Intensive Care Unit, Duitama Regional Hospital, Duitama, COL; 2 Research Group - Knowledge Management, Teaching, Development, and Innovation, Duitama Regional Hospital, Duitama, COL

**Keywords:** acute respiratory distress syndrome (ards), co2, lung injury, lung pathophysiology, severe respiratory distress syndrome

## Abstract

We present the case of a young woman with morbid obesity who developed severe acute respiratory distress syndrome (ARDS) associated with influenza A H1N1 infection, who, despite providing volume-controlled ventilation strategies, with a positive end-expiratory pressure (PEEP) of 12 cmH₂O, tidal volume of 8 mL/kg of predicted body weight and sedation for Richmond Agitation-Sedation Scale (RASS) score of -3, persisted with refractory hypoxemia and permissive hypercapnia off targets. As stabilization therapy, the initiated veno-venous hemodiafiltration continued with ECCO₂R and cycles of at least 24 hours of prone positioning while awaiting referral to extracorporeal membrane oxygenation (ECMO). Influenza virus A subtype H1N1 has single-stranded RNA and displays on its surface two essential glycoproteins, hemagglutinin (cell adhesion) and neuraminidase (release of new virions), that, due to a dysregulated response of cytokines and proinflammatory factors coupled with increased viral replication, induce greater lung injury and risk of progression to severe ARDS. This medical condition continues to be a challenge for health personnel, with special difficulty in patients with conditions such as morbid obesity, where physiological parameters and ventilatory strategies may have significant variations. Given the severity of the cases and the lack of immediate ECMO availability, ECCO₂R may be an alternative for supporting and maintaining protective ventilatory measures, as mentioned in the case below.

## Introduction

Acute respiratory distress syndrome (ARDS) is a well-known clinical entity, first described in 1967, whose definition has continuously evolved over time. In 1994, the American Thoracic Society and the European Society of Intensive Care Medicine convened a consensus conference to define and standardize the diagnostic criteria for ARDS, which were subsequently revised and modified in 2012 with the development of the Berlin definition. More recently, the 2024 consensus defines ARDS as an acute, diffuse inflammatory lung injury, triggered by infectious and non-infectious etiologies. It is generally characterized by non-hydrostatic pulmonary edema, associated with increased vascular and epithelial permeability, leading to pulmonary edema and atelectasis, ultimately resulting in loss of aerated lung tissue. Clinically, it manifests with arterial hypoxemia and diffuse radiographic opacities, associated with an increase in intrapulmonary shunt and alveolar dead space, as well as reduced lung compliance [1].

The LUNG SAFE study - the largest contemporary study about ARDS epidemiologic data published to date - was conducted in Northern Ireland in 2016, and it included 459 intensive care units (ICUs) from 50 countries, describing an average mortality of 40% in moderate to severe cases of the disease. According to the study, the main causes of ARDS encompass viral, bacterial, and mycotic pneumonia, tuberculosis, extrapulmonary sepsis, aspiration, non-cardiogenic shock, trauma, blood transfusions, pulmonary contusions, inhalational injuries, drug overdose, pulmonary vasculitis, and broad-area burns. On average, patients require eight days of invasive mechanical ventilation (IMV) and 10 days of ICU stay [2].

Influenza A virus, subtype A H1N1, is a significant cause of viral pneumonia and respiratory failure. It has single-stranded RNA and exhibits two essential surface glycoproteins: hemagglutinin and neuraminidase, which enable cell adhesion and the release of newly formed virions, respectively. Influenza virus infection has been shown to activate and manipulate multiple cellular signaling pathways, including the tyrosine kinase receptor (TRK), thereby inducing interferon- and cytokine-dependent antiviral responses in the host. Ultimately, a dysregulated cytokine response with excessive proinflammatory mediator release, together with augmented viral replication, results in greater pulmonary injury and an increased risk of progression to severe ARDS [3].

In Colombia, epidemiological data about the characteristics of ARDS patients are scarce. Varón-Vega et al. conducted a multi-center study that included six adult ICUs and a cohort of 70 patients, in which pneumonia and extrapulmonary were described as the primary causes of ARDS. The study also identified a mortality rate of 46%, with a median duration of 11 days of IMV and a median of 14 days of ICU length of stay [4].

The ARDS management objective is to reduce lung injury, improve oxygenation with controlled permissive hypercapnia, and reduce the risk of patient self-inflicted lung injury (P-SILI).To reach these goals, multiple fundamental pillars exist, such as the maintenance of elevated positive end-expiratory pressure (PEEP) to prevent alveolar overdistension and collapse. In addition, prolonged recruitment maneuvers exceeding 60 seconds should be avoided, and ventilatory strategies that limit tidal volume (TV) to 4-8 mL/kg of body weight with plateau pressures below 30 cmH₂O should be procured. Prone-position ventilation for more than 12 hours per day is recommended as a key therapeutic strategy, particularly in patients with severe ARDS [5]. For those cases with refractory hypoxemia, in particular when there is an impossibility to maintain open-lung ventilatory mechanics, extracorporeal membrane oxygenation (ECMO) therapy is considered [6].

Refractory hypoxemia, in the context of ARDS, is associated with high mortality, which is related to the multi-organ dysfunction it provokes. Low lung compliance and marked limitations in ventilation (TV 4-8 mL/kg of predicted body weight and plateau pressure (PPLAT) <30 cmH₂O) due to reduced distensibility help explain severe hypoxemia. Lung-protective ventilation strategies may lead to secondary hypercapnia, which in turn results in undesirable neurologic and cardiovascular effects. In these circumstances, CO₂ removal through a veno-venous extracorporeal circuit is an alternative utilized in selected cases; however, data in patients with morbid obesity remain limited, despite its emergence as a potential alternative or adjunct to ECMO [7]. Although ECMO represents an effective strategy in such scenarios, its availability in Colombia is restricted to high-complexity centers. This case reports the utilization of continuous veno-venous hemofiltration (CVVH) with a CO₂ removal filter, coupled with lung-protective mechanical ventilation and prolonged prone positioning, in a patient with morbid obesity while awaiting veno-venous ECMO.

## Case presentation

A 26-year-old woman, with no known medical comorbidities and a history of morbid obesity (BMI 49 kg/m²), presented to a medium-complexity hospital in Colombia with a three-day clinical presentation of malaise, emesis-inducing cough, nasal congestion, and dyspnea. She also reported three episodes of non-mucoid and non-blood diarrhea, with no fever and no gastrointestinal or urinary symptoms. During physical examination, she was afebrile (36°C), normotensive (130/80 mmHg), hypoxemic (SpO₂ 85%), and tachycardic (heart rate 125 bpm). Cardiac auscultation was normal, and pulmonary examination revealed bilateral expiratory wheezing, associated with diminished vesicular breath sounds.

**Figure 1:**
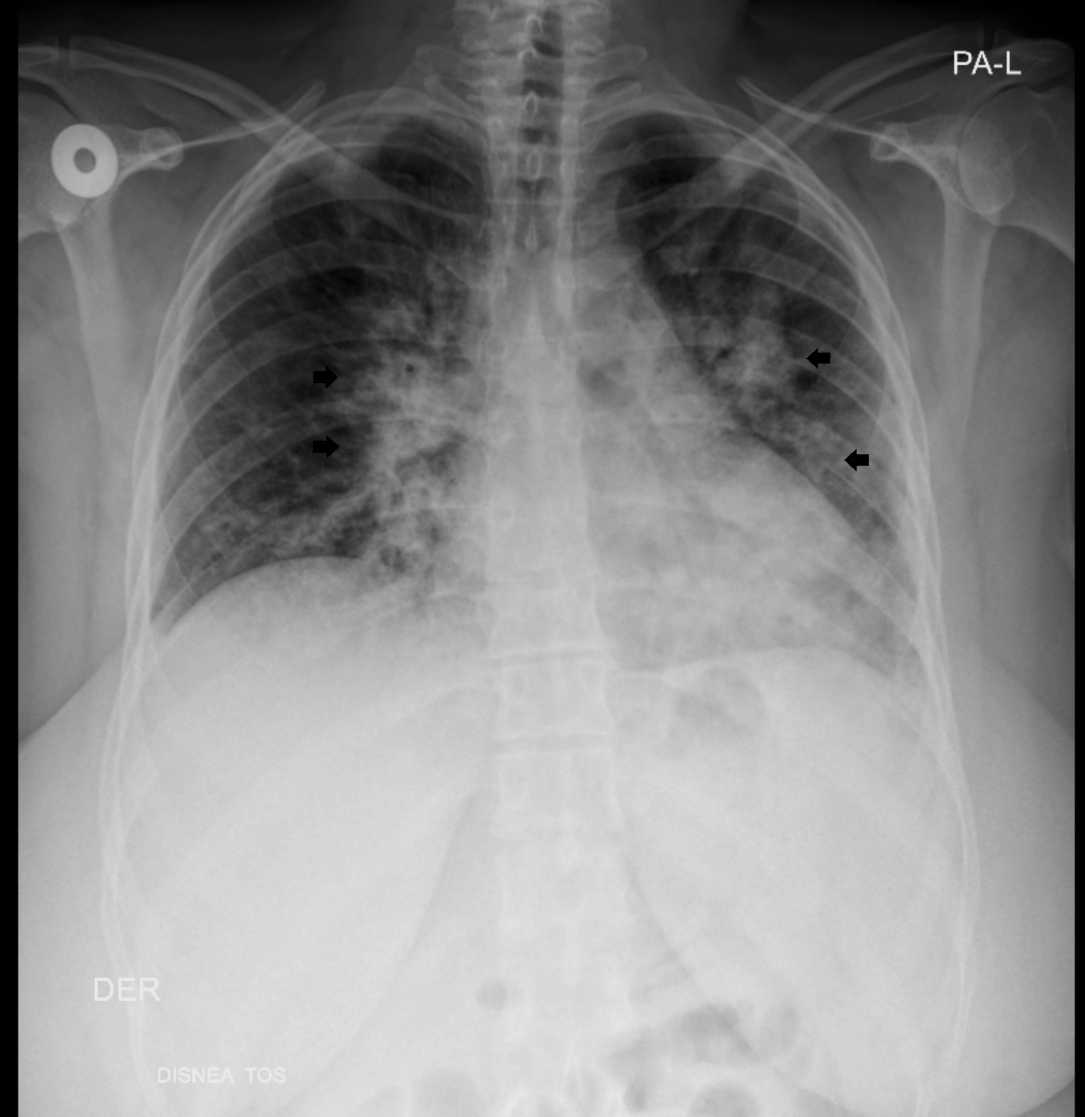
Chest X-ray: with diffuse alveolo-interstitial infiltrate predominantly on the left. Black arrows: increased pulmonary radiolucency due to diffuse alveolar-interstitial infiltrate.

After evaluation in the emergency department, where hospital admission was indicated, and treatment for a presumed acute upper respiratory tract infection of bacterial origin was initiated, with aminopenicillins, intravenous corticosteroids, and supplemental oxygen via nasal canula. Hospital admission laboratory tests showed a normal complete blood count, a markedly elevated C-reactive protein, no electrolyte disorders, normal renal and thyroid function, and an elevated glycated hemoglobin. SARS-CoV-2 testing was negative. Chest radiography revealed diffuse alveolar-interstitial infiltrates (Figure 1).

**Figure 2:**
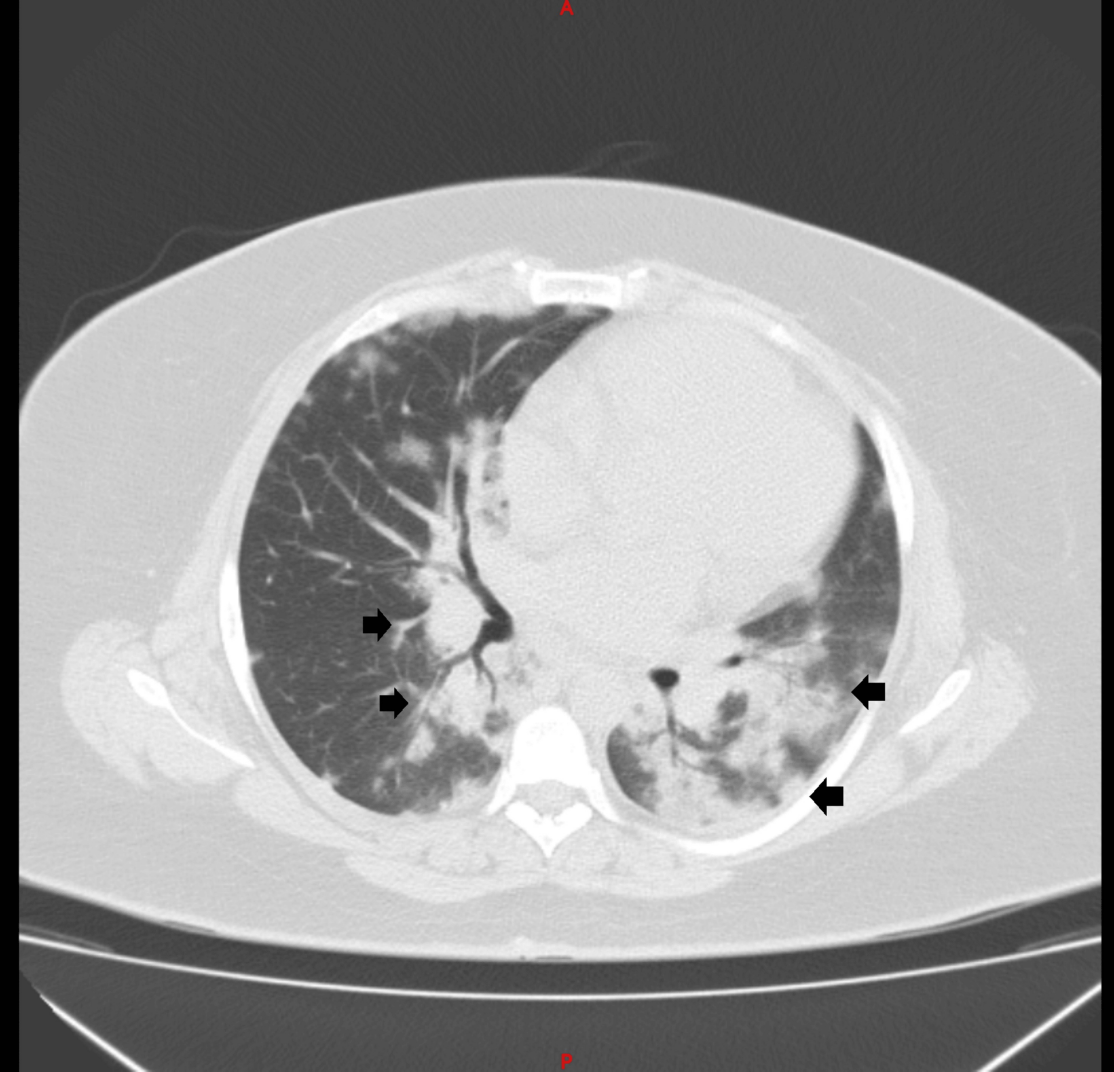
Computed tomography axial slice: air bronchogram with central and peripheral involvement, with approximately 40% of the lung parenchyma compromised. Black arrows: multiple alveolar opacities are observed in the lung parenchyma.

**Figure 3:**
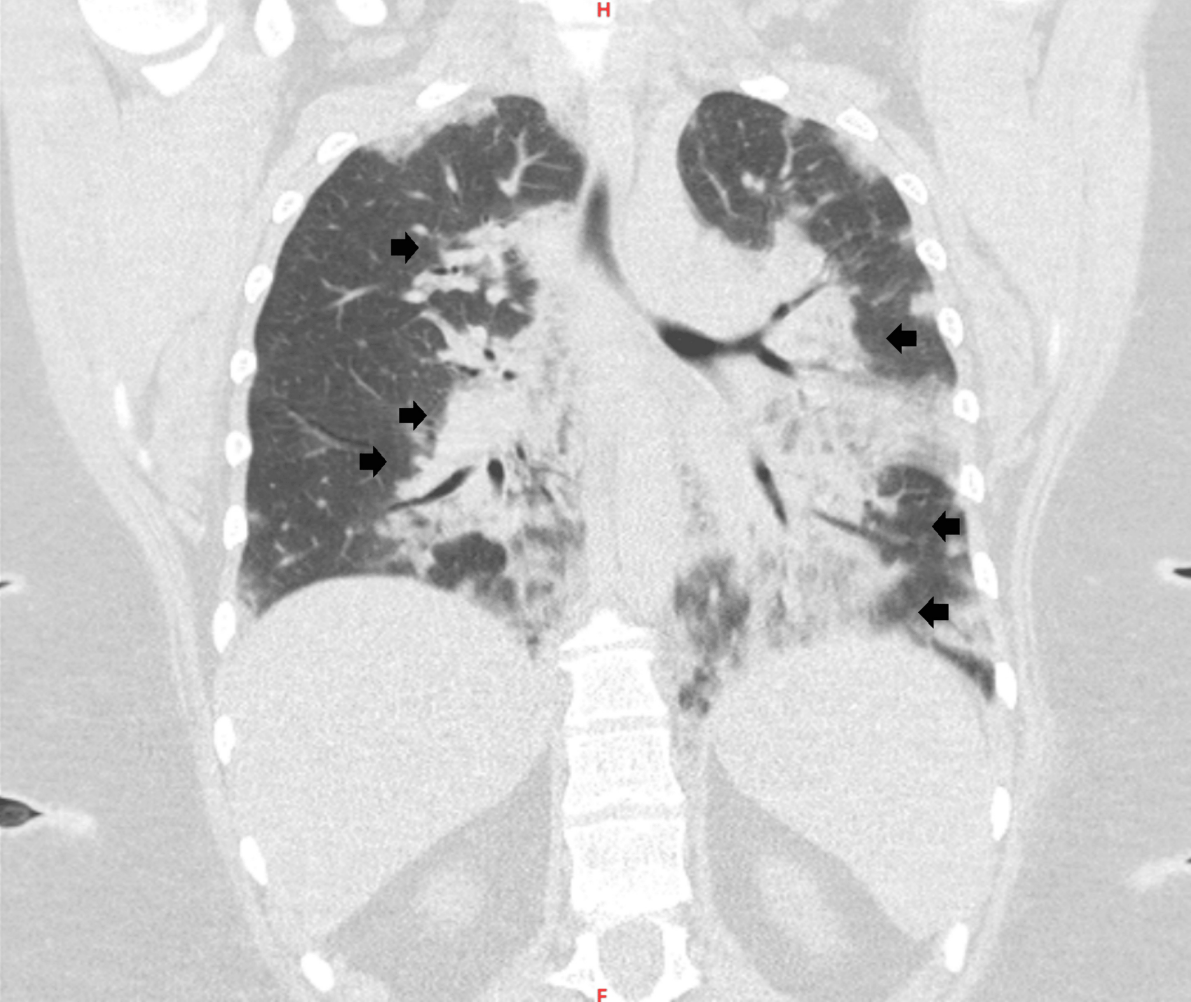
Computed tomography coronal slice: air bronchogram with central and peripheral involvement, with approximately 40% of the lung parenchyma compromised. Black arrows: multiple alveolar opacities are observed in the lung parenchyma.

During her hospital stay, she presented with persistent respiratory symptoms, including fever and hemoptysis, which resulted in resistance to initial antibiotic therapy. Consequently, escalation to a fourth-generation cephalosporin in combination with a macrolide was ordered. Additionally, a chest computed tomography (CT) scan was obtained, displaying alveolar opacities and air bronchogram, with central and peripheral lung involvement, compromising approximately 40% of the pulmonary parenchyma (Figures 2-3).

A respiratory pathogen panel detected influenza A H1N1 (PDM09), and GeneXpert testing ruled out *Mycobacterium* infection. Oseltamivir was added to her management plan; however, in the face of increased oxygen requirements, she progressed to type I respiratory failure, with severe pulmonary dysfunction (PaO₂/FiO₂ ratio 52). In light of the clinical deterioration, orotracheal intubation was performed, and she was transferred to the ICU.

At ICU admission, she required vasopressor support due to hypotension refractory to intravenous fluid resuscitation, without signs of tissue hypoperfusion, indicated by normal serum lactate and capillary refill time. Arterial blood gas analysis showed a PaCO₂ of 52 mmHg and a PaO₂ of 42 mmHg, with a PaO₂/FiO₂ ratio of 42. These findings were associated with elevated airway pressures: peak inspiratory pressure (PIP) of 40 cmH₂O, plateau pressure (Pplat) of 35 cmH₂O, and a driving pressure (DP) of 23 cmH₂O. Airway resistance was 20 cmH₂O/L/s, with dynamic compliance 19 mL/cmH₂O and static compliance 28 mL/cmH₂O. 

Despite a volume-controlled ventilation configuration with PEEP 12 cmH₂O, a TV of 8 mL/kg of predicted body weight (56 kg), and sedation targeting a Richmond Agitation-Sedation Scale (RASS) score of 3, persistent refractory hypoxemia was present, and the patient was classified as having severe ARDS. Consequently, neuromuscular blockade with cisatracurium was initiated, and the patient was placed in the prone position. Although she presented an improvement in oxygenation indices, permissive hypercapnia and airway pressures remained out of target, hence why, given the severity of the illness, referral procedures for ECMO therapy were initiated.

While waiting for referral, CVVHDF and ECCO₂R therapy were initiated trough the insertion of a high-flow catheter in the right anterior jugular vein, in accordance with the following parameters and laboratory monitoring (Table 1): blood flow of 200 mL/min, dialysate flow 2 L/hour, membrane surface area 0.8 m², sweep gas flow 2.5 L/min and anticoagulation with unfractioned heparin at 1 units/kg/hour of predicted bodyweight dose. The latter approach was undertaken in the setting of a worsening of renal function and a cumulative fluid balance of 8000 cc. It was combined with prone positioning cycles of at least 24 hours. In parallel, estimating that the patient could present increased chest wall elastance, a higher PEEP was used (16 cmH₂O), allowing for an objective improvement of ventilatory mechanics and oxygenation parameters.

**Table 1:** Summary of paraclinical tests. aPTT: activated partial thromboplastin time; BUN: blood urea nitrogen; Ca: calcium; Cl: chloride; Cr: creatinine; Hct: hematocrit; Hgb: hemoglobin; INR: international normalized ratio; K: potassium; Lymp: lymphocytes; Mg: magnesium; Na: sodium; Neu: neutrophils; Plt: platelets; PT: prothrombin time; WBC: white blood cells Additional laboratory findings: glycated hemoglobin: 6.0%; total cholesterol: 61 mg/dL; HDL cholesterol: 15 mg/dL; LDL cholesterol: 20 mg/dL; triglycerides: 133 mg/dL; thyroid-stimulating hormone: 0.5 µIU/mL; free thyroxine (T4): 1.33 ng/dL. *ECCO₂R startup reference values

Day	Cr (mg/dL)	BUN (mg/dL)	Na (mmol/L)	K (mmol/L)	Cl (mmol/L)	Mg (mmol/L)	Ca (mmol/L)	WBC (µL)	Neu (%)	Lymp (%)	Hgb (g/dL)	Hct (%)	Plt (µL)	PT (sec)	aPTT (sec)	INR
Ref.	0.5-1.04	6-20	135-145	3.5-5.2	96-106	1.7-2.2	1.1-1.3	4500-11000	40-75	20-45	11.6-15	36-48	150,000-450,000	11-14	25-35	0.8-1.2
1	0.64	7.26	142	3.7	106	-	-	2750	83.2	15.4	12.5	39.0	133,000	11.0	33.1	1.1
2*	1.20	12.50	145	4.5	-	-	-	9130	86.5	10.5	13.5	44.0	151,000	10.4	26.9	1.04
3	0.76	9.30	145	3.9	108	2.54	0.90	8250	73.9	15.5	13.0	41.6	134,000	13.0	117.0	1.3
4	1.70	14.00	144	4.7	108	2.54	0.90	16130	77.0	11.0	13.9	44.2	201,000	14.0	26.9	1.04
5	1.50	16.00	144	4.9	108	1.90	0.90	19580	79.0	7.8	11.7	37.4	204,000	10.9	49.0	1.09
6	1.80	17.00	142	5.4	106	1.90	0.95	19580	79.0	7.8	11.7	37.4	204,000	-	-	-
7	1.80	18.00	143	5.3	106	-	-	19470	77.0	6.6	10.0	33.3	176,000	10.4	32.0	1.04
8	1.79	18.90	143	4.7	107	2.70	0.90	16610	79.0	3.6	9.7	31.2	177,000	-	-	-

**Figure 4:**
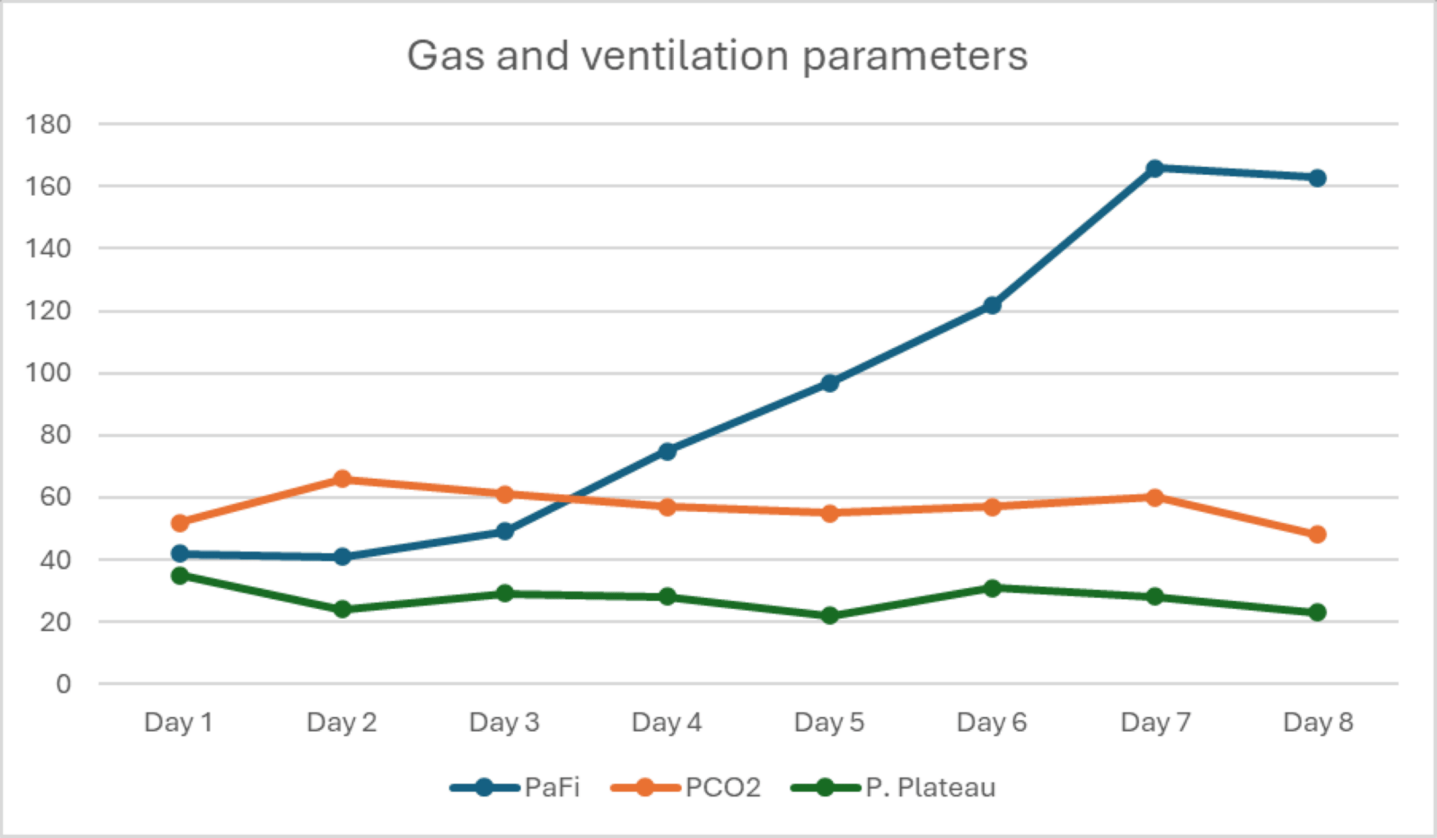
Ventilatory and gasometric trends over time.

Upon day 1 after ECCO₂R instauration, the DP was maintained at ≤15 cmH₂O. On day 2, DP was in the range of 10 cmH₂O, and on day 3, it ranged between 10 and 15 cmH₂O along with VT set between 7 and 7.5 mL/kg of predicted bodyweight, in an effort to maintain lung-protective ventilation strategies; however, it is important to clarify that delta DP fluctuated due to clinical instability and difficulty when attempting to reduce ventilatory volumes, which was associated with sustained desaturation in unacceptable ranges (<50%), accompanied with hemodynamic instability (Table 2 and Figure 4).

**Table 2:** Arterial and venous blood gases and respiratory mechanics. ΔCO₂: central venous-to-arterial carbon dioxide tension difference; HCO₃⁻: bicarbonate; P. Peak: peak inspiratory pressure; P. Plateau: plateau pressure; PaCO₂: partial pressure of carbon dioxide; PaO₂: partial pressure of oxygen; PaO₂/FiO₂: ratio of partial pressure of arterial oxygen to fraction of inspired oxygen; PEEP: positive end-expiratory pressure; S. No.: serial number; SatO₂: oxygen saturation; ScvO₂: central venous oxygen saturation; Static Comp.: static compliance (distensibility); TV: tidal volume

S. No.	Day	Position/Strategy	pH	PaCO₂ (mmHg)	PaO₂ (mmHg)	SatO₂ (%)	HCO₃⁻ (mEq/L)	PaO₂/FiO₂	ScvO₂ (%)	ΔCO₂ (mmHg)	O₂ Extraction (%)	TV (mL)	PEEP (cmH₂O)	P. Peak (cmH₂O)	P. Plateau (cmH₂O)	Static Comp. (mL/cmH₂O)	Driving Pressure (cmH₂O)
1	1	Admission - Supine	7.30	52	42	75	22	42	62	2	17	450	12	40	35	28	23
2	1	Prone	7.23	52	39	68	19	39	56	9	17	450	14	33	29	26	15
3	2	Prone + ECCO₂R	7.16	66	45	69	18	41	58	5	15	450	14	30	24	37	10
4	3	Prone	7.29	61	49	82	22	49	70	6	14	420	14	41	29	24	15
5	4	Supine	7.27	55	30	57	21	30	50	5	11	420	14	40	33	28	19
6	5	Prone	7.25	55	58	87	21	97	74	5	15	450	14	34	22	44	8
7	6	Prone	7.26	57	61	90	22	122	-	-	-	450	14	41	31	31	17
8	7	Supine	7.23	60	83	95	22	166	84	7	11	400	12	39	28	34	16
9	8	Prone	7.31	48	57	89	23	163	74	5	16	450	12	34	23	40	11

After 15 consecutive days of ECCO₂R therapy and without achieving access to an ECMO-capable center due to lack of availability and transfer difficulties, the patient developed fibrotic changes of the pulmonary parenchyma with persistent requirement for IMV, high inspired oxygen fractions (FiO₂ > 80%), and severe refractory hypoxemia (PaO₂ < 50 mmHg). After 74 days in the ICU, the patient died.

## Discussion

ECMO therapy is the treatment of choice in cases such as the one reported; nevertheless, this technological resource and the specialized personnel are often not accessible in medium- and low-complexity hospitals. On the other hand, geographical barriers, such as those existing in Colombia, pose a significant challenge for the management of ARDS in provincial settings, given the high mortality associated with the disease. The strategy herein presented - continuation of ultraprotective IMV, prolonged prone positioning, and veno-venous circuit CO₂ removal - can be used in similar settings while access to an ECMO-capable center is achieved.

While ECCO₂R may mitigate hypercapnia associated with lung-protective ventilation and increased dead space, its use has been linked to higher rates of hemorrhagic and hematologic complications. In the REST trial, no mortality benefit was demonstrated despite an improvement in ventilator-free days, underscoring that its potential value likely hinges on meticulous, physiology-driven patient selection [8]. On the other hand, Acharta et al. [9] described a patient with severe ARDS secondary to influenza, without obesity, in whom ECCO₂R was implemented using a controlled arteriovenous (A-V) technique within the first 48 hours after diagnosis, resulting in clinical stabilization and resolution of the condition. According to the authors, no ECCO₂R-related complications were observed, and the therapy was discontinued after 12 days of use; however, it is important to note that this was only a case report.

Gracitúa et al. [10] described a report of a young, overweight patient with severe ARDS secondary to COVID-19, in whom, due to severe hypercapnia, a veno-venous ECCO₂R filter was implemented using a pre-dialysis configuration, a technique similar to that used in the present case. In the case reported by Gracitúa et al., ECCO₂R was initiated after 21 days of mechanical ventilation start. After 27 days of ECCO₂R therapy use, the patient exhibited satisfactory clinical evolution and hospital discharge [10]. 

None of the previously reported cases in the medical literature included patients with severe ARDS secondary to influenza, in which ECCO₂R was documented, who had a history of morbid obesity, with the physiological implications it has on the biophysics of mechanical ventilation and on carbon dioxide production and elimination. The contribution of this case report to the scientific community lies in the possibility of successful utilization of ECCO₂R in patients with morbid obesity (BMI: 49 kg/m²).

From a different standpoint, ECCO₂R support counteracts hypercapnia efficiently and facilitates low-volume mechanical ventilation. As reported by Monet et al. in a retrospective study involving 45 ECCO₂R sessions, in which a 40% reduction of TV -up to ≤ 3 ml/kg of predicted body weight-was achieved, particularly with a high-flow technique, a finding that may be associated with better clinical outcomes [7].

Finally, ECCO₂R is an extracorporeal support technique whose implementation methods have changed over time. Currently, the veno-venous catheter technique is preferred; its main indications include both hypoxemic and hypercapnic acute respiratory failure associated with chronic obstructive pulmonary disease (COPD), severe asthma, and medical management refractory respiratory acidosis. Overall, if a patient is a candidate to receive ECMO therapy, therapeutic instauration should not be delayed; nonetheless, CO₂ levels exceeding permissive hypercapnia can be controlled through ECCO₂R use [6]. 

These suggestions should be substantiated by further prospective studies, preferably with a randomized controlled design, to address cofounding factors and contribute to answering questions that may arise from this case report.

## Conclusions

In patients with severe ARDS due to viral pneumonia secondary to A (H1N1) influenza virus infection, who are managed with lung-protective ventilation in prone positioning but develop arterial CO₂ values exceeding permissible thresholds, although ECCO₂R may mitigate the cardiovascular and metabolic complications of severe hypercapnia and facilitate mechanical ventilation, these physiological benefits have not translated into improved clinical outcomes. Its role in resource-limited settings, however, may be relevant as a bridge to ECMO therapy to gain time. This case is of particular interest due to the history of morbid obesity (BMI: 49 kg/m²) associated with it, which further complicates mechanical inter-hospital transfer by ground ambulance under mechanical ventilation across long distances. 
